# Tumor-infiltrating lymphocyte enrichment predicted by CT radiomics analysis is associated with clinical outcomes of non-small cell lung cancer patients receiving immune checkpoint inhibitors

**DOI:** 10.3389/fimmu.2022.1038089

**Published:** 2023-01-05

**Authors:** Changhee Park, Dong Young Jeong, Yeonu Choi, You Jin Oh, Jonghoon Kim, Jeongun Ryu, Kyunghyun Paeng, Se-Hoon Lee, Chan-Young Ock, Ho Yun Lee

**Affiliations:** ^1^ Department of Internal Medicine, Seoul National University Hospital, Seoul, Republic of Korea; ^2^ Department of Radiology, Samsung Medical Center, Sungkyunkwan University School of Medicine, Seoul, Republic of Korea; ^3^ Department of Health Sciences and Technology, Samsung Advanced Institute for Health Sciences & Technology (SAIHST), Sungkyunkwan University, Seoul, Republic of Korea; ^4^ Department of Electronic and Computer Engineering, Sungkyunkwan University, Suwon, Republic of Korea; ^5^ Lunit, Seoul, Republic of Korea; ^6^ Division of Hematology Oncology, Department of Medicine, Samsung Medical Center, Sungkyunkwan University School of Medicine, Seoul, Republic of Korea

**Keywords:** radiomics, immune checkpoint inhibitor (ICI), immunotherapy, tumor infiltrating lymphocyte (TIL), artificial intelligence

## Abstract

**Background:**

Enrichment of tumor-infiltrating lymphocytes (TIL) in the tumor microenvironment (TME) is a reliable biomarker of immune checkpoint inhibitors (ICI) in non-small cell lung cancer (NSCLC). Phenotyping through computed tomography (CT) radiomics has the overcome the limitations of tissue-based assessment, including for TIL analysis. Here, we assess TIL enrichment objectively using an artificial intelligence-powered TIL analysis in hematoxylin and eosin (H&E) image and analyze its association with quantitative radiomic features (RFs). Clinical significance of the selected RFs is then validated in the independent NSCLC patients who received ICI.

**Methods:**

In the training cohort containing both tumor tissue samples and corresponding CT images obtained within 1 month, we extracted 86 RFs from the CT images. The TIL enrichment score (TILes) was defined as the fraction of tissue area with high intra-tumoral or stromal TIL density divided by the whole TME area, as measured on an H&E slide. From the corresponding CT images, the least absolute shrinkage and selection operator model was then developed using features that were significantly associated with TIL enrichment. The CT model was applied to CT images from the validation cohort, which included NSCLC patients who received ICI monotherapy.

**Results:**

A total of 220 NSCLC samples were included in the training cohort. After filtering the RFs, two features, gray level variance (coefficient 1.71 x 10^-3^) and large area low gray level emphasis (coefficient -2.48 x 10^-5^), were included in the model. The two features were both computed from the size-zone matrix, which has strength in reflecting intralesional texture heterogeneity. In the validation cohort, the patients with high predicted TILes (≥ median) had significantly prolonged progression-free survival compared to those with low predicted TILes (median 4.0 months [95% CI 2.2–5.7] versus 2.1 months [95% CI 1.6–3.1], p = 0.002). Patients who experienced a response to ICI or stable disease with ICI had higher predicted TILes compared with the patients who experienced progressive disease as the best response (p = 0.001, p = 0.036, respectively). Predicted TILes was significantly associated with progression-free survival independent of PD-L1 status.

**Conclusions:**

In this CT radiomics model, predicted TILes was significantly associated with ICI outcomes in NSCLC patients. Analyzing TME through radiomics may overcome the limitations of tissue-based analysis and assist clinical decisions regarding ICI.

## Introduction

Treating non-small cell lung cancer (NSCLC) with immune checkpoint inhibitors (ICI) has become a prevailing strategies since the clinical benefits have been demonstrated by numerous clinical trials (1–3). Various studies on the immune tumor microenvironment (iTME) have been conducted to identify patients who would benefit from ICI (4). Some of the most prevalent biomarkers that represent iTME include programmed cell death ligand 1 (PD-L1) expression, tumor mutation burden, and tumor-infiltrating lymphocytes (TILs) (4). However, these biomarkers require tissue biopsy through invasive procedures, which is difficult to perform repeatedly and sometimes even impossible. Radiomics may overcome such limitations as radiologic analysis is much less invasive than a tissue biopsy (5). Radiomics in medicine is the practice of processing high-throughput extraction of quantitative features to convert images such as computed tomography (CT) into mineable data and analyze the data for decision support (6). Studies have demonstrated the association of radiomic features with histologic findings such as histological subtypes of lung cancers (7, 8). Furthermore, radiomic features could represent TME and genomic instability, which have not been demonstrated by current functional imaging (9). Therefore, a radiomic approach in NSCLC may provide spatial information on TIL and thereby assist clinical decisions in the use of ICIs.

Previous studies have demonstrated the feasibilities of this approach. Yoon et al. (10) reported that radiomic features could be potential biomarkers in identifying type 2 helper T (Th2) cell signatures. Sun et al. (11) published the results of a radiomic signature model that predicts CD8 cells based on *CD8B* gene-associated signatures in NSCLC tumors and correlated this with ICI treatment outcomes. Tang et al. (12) reported the development of a PD-L1 and CD3 immunohistochemistry informed radiomics model dividing NSCLC into four clusters that correlated with overall survival. There is also a study on the radiomics of positron emission tomography which developed a deep-learning model predicting a cytolytic activity score that was associated with ICI outcomes and the heterogeneity of responses (13). However, these studies were based on biomarkers that are crude representatives of iTME and not often used in clinical practices. In addition, pathophysiologic insights regarding how radiomic models are associated with iTME have not been compelling enough.

Here, we searched for the radiomic features that are potentially reflective of iTME through the use of TIL. For an objective TIL assessment, we used an artificial intelligence (AI)-powered TIL analyzer, Lunit SCOPE IO, and hematoxylin and eosin (H&E) stained slides (14). The Lunit SCOPE IO determines the immune phenotype of tumors by TIL assessment, which showed a significant association with ICI outcome in advanced NSCLC (14). We identified radiomic features significantly correlated with TIL assessed by Lunit SCOPE IO and validated this data in the ICI treated cohort. For the selected radiomic features, we considered whether the potential pathophysiological mechanism of TIL could be applied to radiomics.

## Methods

**Figure 1:**
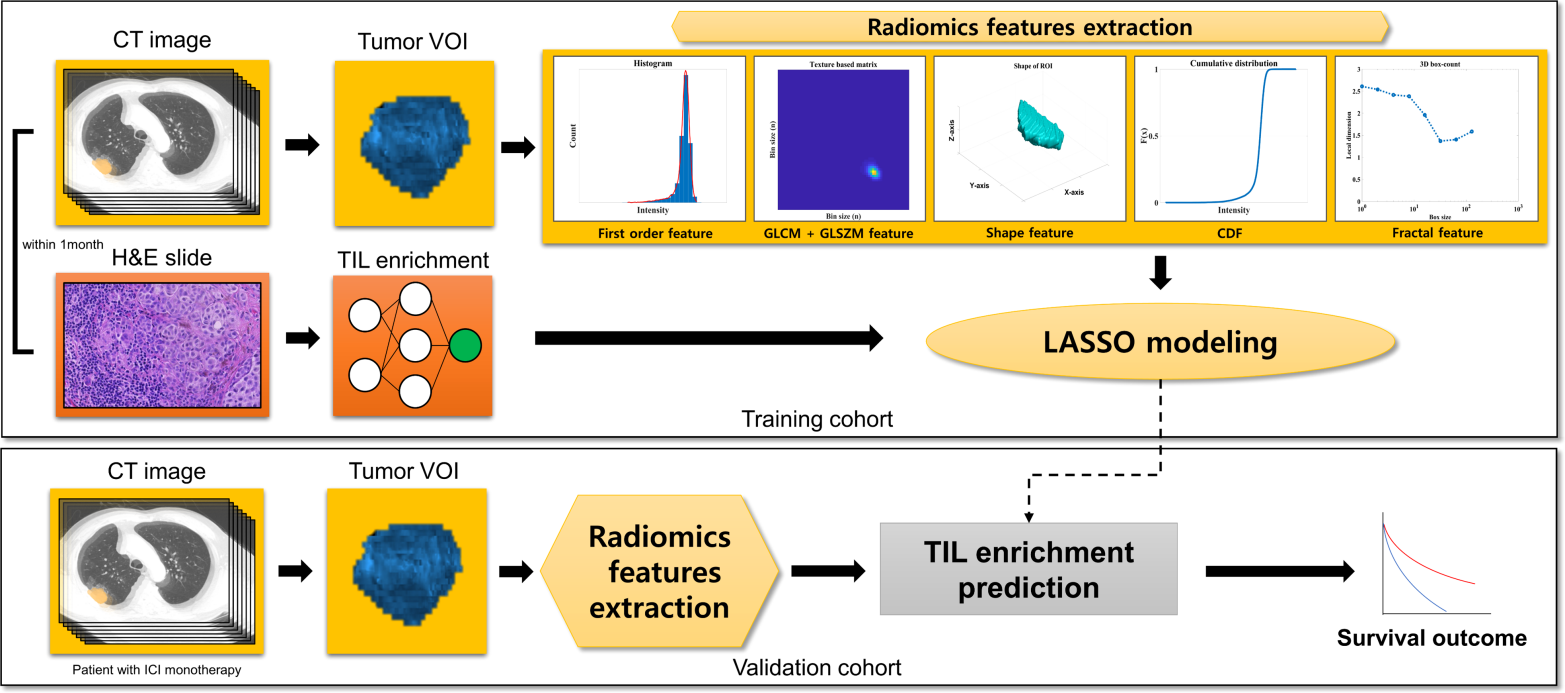
Study scheme.

This is a single-center retrospective cohort study on patients with NSCLC from Samsung Medical Center, Seoul, South Korea. The schematic flow of this study is available in **Figure 1**. We developed least absolute shrinkage and selection operator (LASSO) models from the training cohort predicting the TIL enrichment score (TILes) with radiomic features, which will be described in detail later. Using the model, we calculated the predicted TILes in the validation cohort and evaluated their association with ICI outcomes. The demographic features of the patient, including pathologic diagnosis, *EGFR* mutation, *ALK* translocation, and PD-L1 status, were reviewed. PD-L1 status was defined as high if the tissue showed an ≥50% tumor proportional score (TPS) by PD-L1 immunohistochemistry 22C3 pharmDx and as low if otherwise. The progression-free survival (PFS) and overall survival (OS) of the validation cohort in association with ICIs was also investigated. The response of the disease was determined using revised response evaluation criteria in solid tumors guideline (RECIST) version 1.1 (15).

The study was approved by the institutional review board (IRB) of Samsung Medical Center (IRB number: 2021-04-196). All the data of the current study were collected and analyzed after approval and were in accordance with the declaration of Helsinki.

### Patients of the training and validation cohorts

For the training cohort, the patients who were diagnosed with NSCLC from January 2005 to May 2021 and with available H&E-stained tissue from whole tumor and CT images of the lung acquired within 1 month of each other were included. For the validation cohort, the patients who received ICI monotherapy for advanced NSCLC from January 2013 to May 2021 and with CT images available before 1 year of ICI initiation were included. Patients with CT images not passing the quality for radiomic feature extraction were excluded. (**Supplementary Figure 1**)

### Determining TILes

The version of Lunit SCOPE IO used for this study contains a cell detection AI model and tissue segmentation AI model that were updated from the version described previously in the published article (14). The detailed methods for the development of the Lunit SCOPE IO TIL analyzer are available in **Supplementary Methods 1**.

From the tissues of the training cohort, TILes was defined as the fraction of tissue with high intra-tumoral TIL density (inflamed immune phenotype) or stromal TIL density (immune-excluded immune phenotype) divided by the whole analyzable TME area as measured on the H&E slide. For quality control, samples with less than 0.5 mm^2^ of the cancer epithelium area in the whole H&E slide image and with fewer than ten 1 mm^2^-sized grids available for the evaluation were excluded.

### CT acquisition and radiomic feature extraction

All the patients of the training cohort and the validation cohort underwent contrast-enhanced CT scans using the standardized protocol of our institution. The definition for target lesions were adopted from RECIST version 1.1 (15). The representative target lesions were selected by one thoracic radiologist and one technician (D.Y.J. with 6 years of experience and Y.J.O. with 5 years of experience), reviewed by one senior thoracic radiologist (H.Y.L. with 17 years of experience). Target lesions were segmented by drawing a volume of interest (VOI) with a semiautomatic approach using commercial software AVIEW COPD (version 1.1.38.6, Coreline soft, Seoul, South Korea) and a slice-per-slice approach. Then the boundary of the lesion was modified manually to avoid adjacent air, fat, blood vessels, and surrounding organs. Detailed CT parameters and the 3D segmentation process are also described in **Supplementary Methods 2**.

A total of 88 radiomic features of raw imaging over the given region of interest (ROI) were extracted using a combination of open-source (Pyradiomics, version 3.0.1, Pyradiomics Community) (16) and in-house MATLAB code (MATLAB, R2017, Mathworks Inc., Natick, MA, USA) (17).

The extracted features can be classified into seven categories: (I) first order (intensity) features (n=18); (II) shape features (n=14); (III) gray level co-occurrence matrix features (GLCM; n=24); (IV) gray level size zone matrix features (GLSZM; n=16); (V) cumulative distribution function feature (CDF, n=5); (VI) physical features (n=2); and (VII) Fractal features (n=9). CDF, physical and fractal features were extracted by in-house MATLAB code as they were not calculated in PyRadiomics. A detailed definition of the features is explained in **Supplementary Table 1**.

### Feature selection associated with TILes

In the process of feature selection, we first searched for features that have significantly different TILes values. For each feature, samples were divided into high and low groups by the median value of the feature. Samples with the median value of the feature were classified into the high group. Then, Student’s t test was performed to evaluate the significance of the difference in TILes between the high and low groups of each feature. The cutoff of p< 0.005 was used to filter out and select significant features.

After the filtering process, logistic regressions were performed using TILes as the dependent variable and the selected features as the independent variables. The aliasing features were excluded. Then, the variance inflation factors were calculated to exclude the features showing multicollinearity, which was determined with a variance inflation factor value of more than 10. With the final remaining features, LASSO modeling was performed and features with a non-zero coefficient were finally selected. The models were used to predict TILes in the training cohort to compare with the original TILes. The LASSO modeling was performed using “glmnet” package in R statistics, which solves the objective function for the Gaussian family


$$\underset{\left(\beta_{0},\beta \right)\in {\mathbb{R}}^{p+1}}{\min}\frac{1}{2N}\sum_{i=1}^{N}{\left(y_{i}-\beta_{0}-x_{i}^{T}\beta \right)}^{2}+\lambda \left[\frac{\left(1-\alpha \right){∥\beta ∥}_{2}^{2}}{2}+\alpha {∥\beta ∥}_{1}\right]$$

where we have observations *x*_*i*_∈*ℝ* ^*p*^ , the responses *y*_*i*_∈*ℝ* , *i*=1, …, *N* , and *λ*≥0 is a complexity parameter (18).

Using the model for TILes, we calculated the predicted TILes from the CT images of the validation cohort. With the predicted TILes, the patients were divided into high and low groups according to the median value. Patients with the median value of the feature were classified into the high group.

### Other statistical analysis

The correlation of continuous values was reported by Spearman’s rank correlation coefficient (ρ). The comparison of continuous values between groups was analyzed using the Wilcoxon rank-sum test. The comparison of categorical values between groups was analyzed using Fisher’s exact test and reported with an odds ratio (OR). The survival analysis was performed using the log-rank test and visualized by Kaplan-Meier methods. Hazard ratio (HR) and 95% confidence interval (CI) was calculated using a Cox proportional hazard model. A multivariate Cox proportional hazard model was performed on variables with a factor of p< 0.05 in the univariate Cox proportional hazard model. A P-value< 0.05 was considered statistically significant. All the statistical analyses other than described, were performed with R 4.0.0 (https://www.r-project.org/).

## Results

### Patient demographics

A total of 276 patients were eligible for the training cohort. After the quality of the CT image and H&E-stained samples were evaluated, 220 ROIs from 218 patients were included in the training cohort. Two patients had two ROIs because they were associated with two different time points where CT images and matching H&E slides were acquired within 1 month. The other 216 patients had single ROIs. Among the samples of training cohort, 62 specimens came from patients who previously received systemic treatment and 158 specimens came from patients without any previous systemic treatment. For the validation cohort, 430 patients were eligible. After a quality check of the CT images, 294 ROIs from 294 patients were finally included in the validation cohort. A summary of the patients’ demographics is available in **Table 1**.

**Table 1:** Demographic characteristics of samples included in the study.

	Training cohort (N = 220)	Validation cohort (N = 294)
Age, years		
<60	79 (35.9%)	125 (42.5%)
≥60	141 (64.1%)	169 (57.5%)
Sex		
Female	49 (22.3%)	78 (26.5%)
Male	171 (77.7%)	216 (73.5%)
Smoking		
Never	59 (26.8%)	81 (27.6%)
Former	77 (35.0%)	112 (38.1%)
Current	83 (37.7%)	101 (34.4%)
NA	1 (0.5%)	0 (0%)
Specimen type		
Needle biopsy	119 (54.1%)	Not applicable
EBUS-TBNA	29 (13.2%)	Not applicable
Surgical excision	72 (32.7%)	Not applicable
Pathology		
ADC	127 (57.7%)	184 (62.6%)
SqCC	72 (32.7%)	92 (31.3%)
Sarcomatoid	3 (1.4%)	5 (1.7%)
Large cell	4 (1.9%)	2 (0.7%)
Other	14 (6.4%)	11 (3.7%)
AJCC 8^th^ Staging		
I, II	39 (17.7%)	0 (0%)
III	41 (18.6%)	0 (0%)
IV	140 (63.6%)	294 (100%)
*EGFR* status		
Positive	28 (12.7%)	43 (14.6%)
Negative	178 (80.9%)	232 (78.9%)
NA	14 (6.4%)	19 (6.5%)
*ALK* status		
Positive	3 (1.4%)	5 (1.7%)
Negative	201 (91.4%)	270 (91.8%)
NA	16 (7.3%)	19 (6.5%)
PD-L1 status		
High (≥ 50%)	105 (47.7%)	123 (41.8%)
Intermediate (1-49%)	64 (29.1%)	55 (18.7%)
Low (< 1%)	51 (23.2%)	57 (19.4%)
NA	0 (0%)	59 (20.1%)
Line of ICI treatment		
1^st^	Not applicable	50 (17.0%)
2^nd^	Not applicable	135 (45.9%)
3^rd^ or more	Not applicable	109 (37.1%)

ADC, adenocarcinoma; AJCC, American Joint Committee on Cancer; ALK, anaplastic lymphoma kinase; EBUS-TBNA, endobronchial ultrasound guided transbronchial needle aspiration; EGFR, epidermal growth factor receptor; ICI, immune checkpoint inhibitors; NA, not available; PD-L1, programmed death-ligand 1; SqCC, squamous cell carcinoma.

### Significant radiomic features associated with TILes

**Figure 2:**
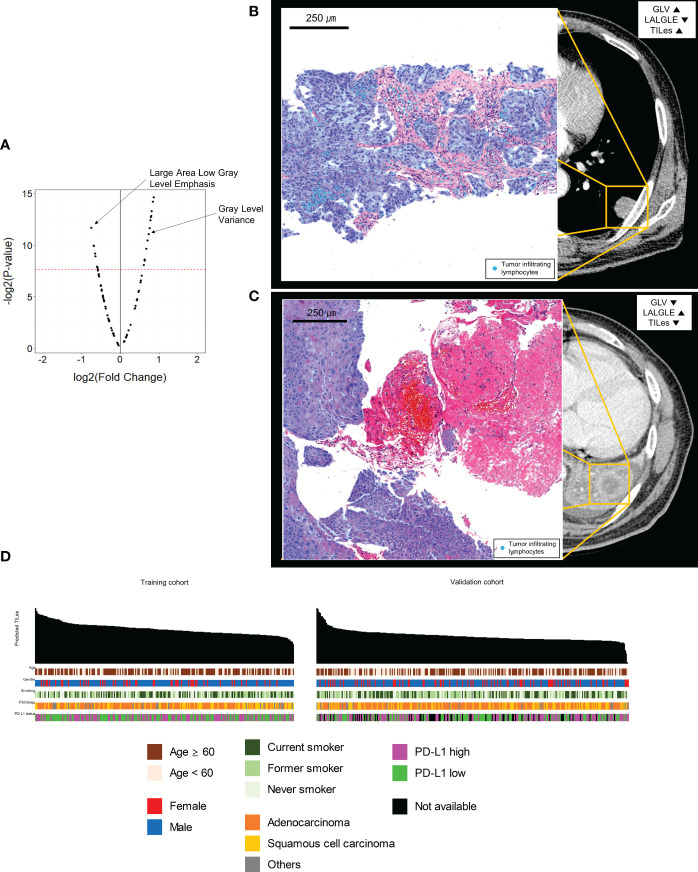
Features associated with tumor infiltrating lymphocyte enrichment score (TILes). **(A)** Volcano plot showing the features associated with TILes. Red horizontal dashed line represents a p-value of 0.005. The points representing Gray Level Variance (GLV) and Large Area Low Gray Level Emphasis (LALGLE) are indicated by arrows. **(B)** CT image and H&E slide example of patient with high GLV and low LALGLE. The blue areas in the H&E slide represent cancer epithelium; the skyblue dots in the H&E slide represent TILs. **(C)** CT image and H&E slide example of patient with low GLV and high LALGLE. The blue areas in the H&E slide represent cancer epithelium; the skyblue dots in the H&E slide represent TILs. **(D)** Bar plots and heatmap showing the distribution of predicted TILes and demographics in the training cohort and validation cohort. The patients are arranged by predicted TILes values in decreasing order in each cohort.

The 88 radiomic features from the 220 ROIs were extracted (**Supplementary Table 2**). After filtering, the radiomic features and modeling process were applied as described in Methods (**Supplementary Table 3**), we found significant features potentially associated with the TILes (**Figure 2A**, **Table 2**). Notably, although the size parameters (maximum 2D diameter slice, maximum 2D diameter column) showed that the larger tumors were associated with lower TILes, these parameters showed multicollinearity and were excluded from the model. Eventually, the LASSO model predicting TILes consisted of two features (**Supplementary Figure 2**), gray level variance (GLV, coefficient 1.71 × 10^-3^) and large area low gray level emphasis (LALGLE, coefficient -2.48 × 10^-5^), which were both GLSZM features. The higher GLV and the lower LALGLE feature values were associated with higher TILes (**Figures 2B, C**). The predicted values by the application of the model in the training cohort significantly correlated with the original values (**Supplementary Figure 3**). The distribution of demographics according to predicted TILes is available in **Figure 2D**.

**Table 2:** The selected significant features associated with TILes.

Number of significantly different features	Number of features finally selected	Name of features	LASSO Coefficient	TILes log2 fold change between feature-high and -low group	TILes difference p-value
22	2	Gray Level Variance	1.71 × 10^-3^	0.732	<0.001
		Large Area Low Gray Level Emphasis	-2.48 × 10^-5^	-0.754	<0.001

**Figure 3:**
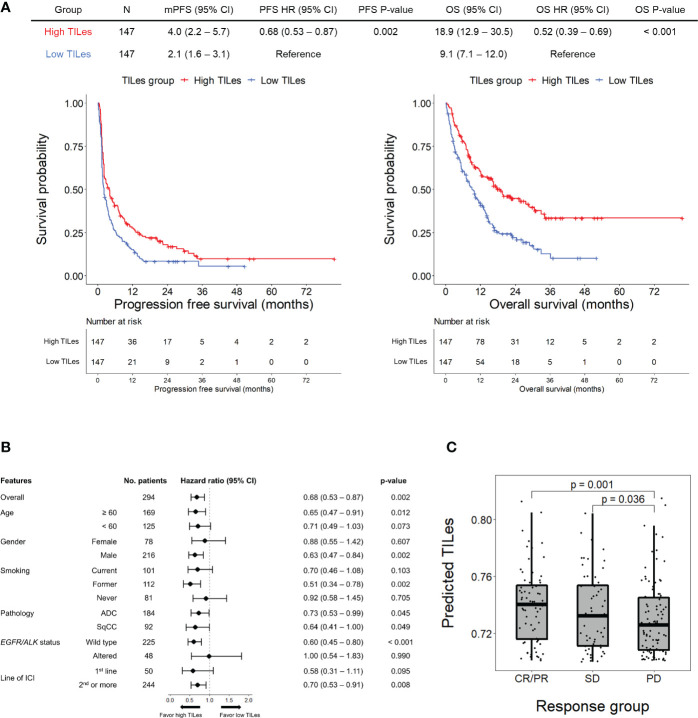
Outcomes of patients receiving immune checkpoint inhibitors (ICI) according to predicted TILes in the validation cohort **(A)** The upper table summarizes the survival analyses of the patients. The left Kaplan-Meier curves show progression free survival (PFS) and the right Kaplan-Meier curves show overall survival (OS) according to the predicted tumor infiltrating lymphocyte enrichment score (TILes) group. The red line represents the high TILes and the blue line represents the low TILes groups. The censored data are marked with vertical lines. The numbers at risk are provided below. **(B)** Forest plot for subgroup analysis of PFS according to predicted TILes in the validation cohort **(C)** The boxplot showing predicted TILes according to the response to ICI. Each dot represents each patient.

Using the LASSO models developed by the selected features, we analyzed the outcome of patients who received ICI in the validation cohort to demonstrate that the model represents the immune aspects of the TILes. We found that patients with high predicted TILes (≥median) show significantly prolonged PFS compared to patients with low predicted TILes (median 4.0 months [95% CI 2.2–5.7] versus 2.1 months [95% CI 1.6–3.1], hazard ratio 0.68 [95% CI 0.53–0.87], p = 0.002; **Figure 3A**). Subgroup analysis generally showed prolonged PFS in the high TILes group compared with that in the low TILes group (**Figure 3B**). In particular, subgroup analysis by pathologic diagnosis showed that patients with high predicted TILes show significantly prolonged PFS compared with patients with low predicted TILes in both adenocarcinoma and squamous cell carcinoma (p = 0.045 and p = 0.049, respectively; **Supplementary Figure 4A**). High predicted TILes also showed significantly prolonged OS compared with patients with low predicted TILes (median 18.9 months [95% CI 12.9–30.5] versus 9.1 months [95% CI 7.1–12.0], hazard ratio 0.52 [95% CI 0.39–0.69], p< 0.001; **Figure 3A**). In addition, patients who experienced a response to ICI or stable disease with ICI had higher predicted TILes compared with the patients who experienced progressive disease as the best response (p = 0.001, p = 0.036, respectively; **Figure 3C**).

**Figure 4:**
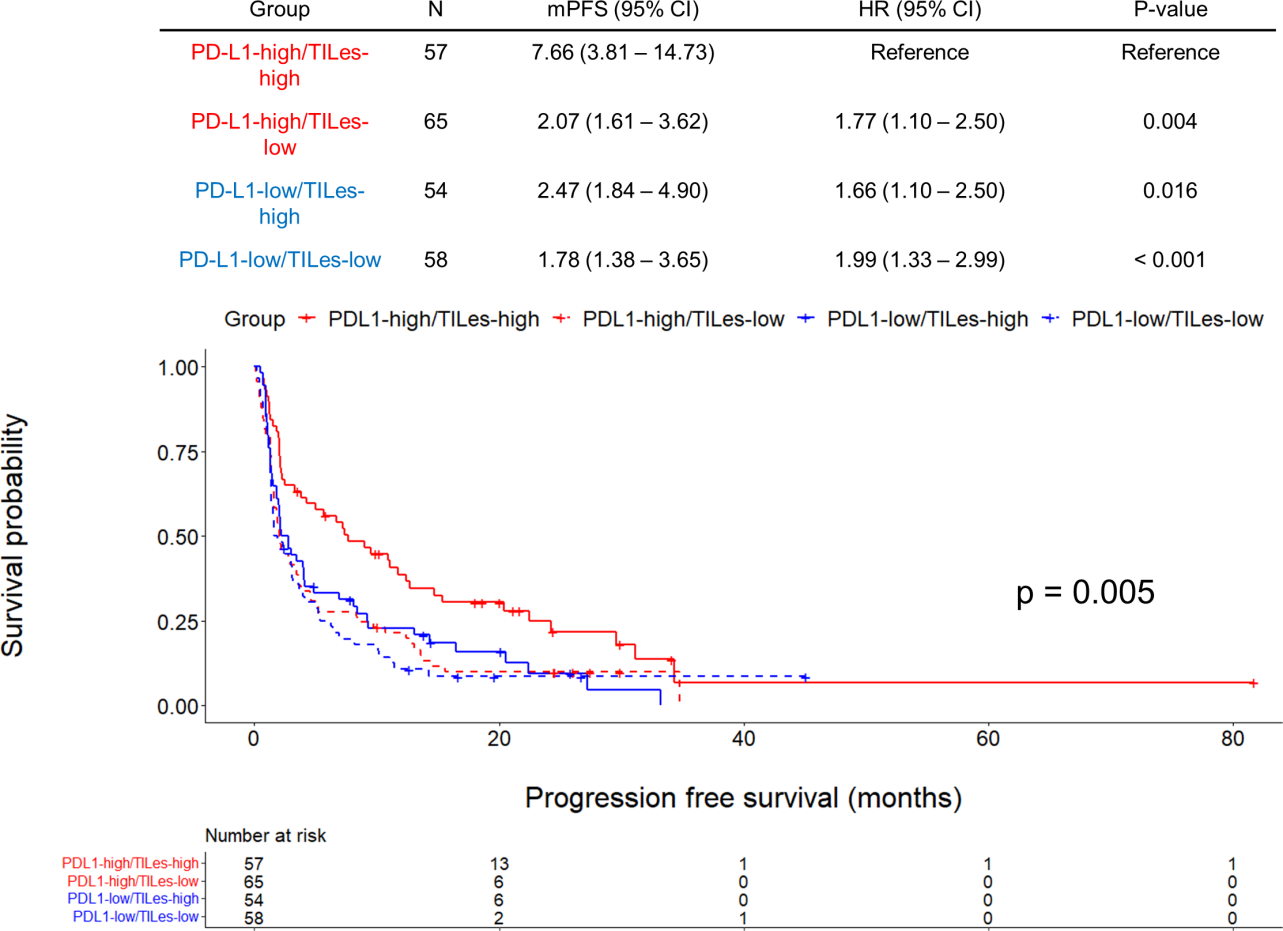
Outcomes of ICI according to predicted tumor infiltrating lymphocyte enrichment score (TILes) and PD-L1 status in the validation cohort. Kaplan-Meier curves showing progression free survival (PFS) according to predicted TILes group and PD-L1 status. Red lines represent the high PD-L1 group and the blue line represents the low PD-L1 group. The solid lines represent the high TILes group and the dashed lines represent the low TILes group. The censored data are marked with vertical lines. The numbers at risk are provided below.

We carried out Cox proportional hazard model analyses to evaluate whether TILes are independent of PD-L1 status, which is well known to be associated with PFS in patients who received ICI as in this cohort (**Supplementary Figure 4B**) and previous studies. We found that predicted TILes was significantly associated with PFS independent of PD-L1 status (HR 0.01, 95% CI 0.00–0.28, p = 0.007 for TILes as continuous variables and HR 0.67, 95% CI 0.51–0.89, p = 0.006 for the high TILes group; **Table 3**). In addition, when the patients were divided into 4 groups according to TILes and PD-L1 status, patients with high TILes and PD-L1 showed significantly prolonged PFS compared with the other 3 groups (p = 0.005; **Figure 4**).

**Table 3:** Cox proportional hazards model for PFS of ICI.

	Univariate analysis		Multivariate analysis (TILes)		Multivariate analysis (TILes group)	
	HR (95% CI)	p-value	HR (95% CI)	p-value	HR (95% CI)	p-value
Age group, years						
< 60	Reference	–	–	–	–	–
≥ 60	0.86 (0.68–1.11)	0.247	–	–	–	–
Sex^#^			–		–	
Female	Reference	–	–	–	–	–
Male	0.82 (0.62–1.08)	0.150	–	–	–	–
Smoking						
Never	Reference	–	Reference	–	Reference	–
Former	0.76 (0.56–1.03)	0.074	0.86 (0.60–1.22)	0.392	0.85 (0.60–1.21)	0.364
Current	0.65 (0.48–0.89)	0.007	0.65 (0.46–0.93)	0.018	0.64 (0.45–0.91)	0.012
Pathology						
ADC	Reference	–	–	–	–	–
SqCC	0.99 (0.76–1.30)	0.955	–	–	–	–
Others	0.91 (0.54–1.52)	0.709	–	–	–	–
PD-L1 TPS group						
Low (< 50%)	Reference	–	Reference	–	Reference	–
High (≥ 50%)	0.74 (0.56–0.97)	0.030	0.72 (0.55–0.96)	0.024	0.73 (0.55–0.96)	0.024
TILes value						
TILes	0.006 (0.001^*^–0.121)	<0.001	0.010 (0.001^*^–0.284)	0.007	–	
TILes group						
Low	Reference	–	–	–	Reference	–
High	0.68 (0.53–0.87)	0.002	–	–	0.67 (0.51–0.89)	0.006

^#^Sex was not included in multivariate analysis because of the significant association of male sex with current smoking (p< 0.001).

*These values were less than 0.001. *Note: ADC, adenocarcinoma; ICI, immune checkpoint inhibitors; PD-L1, programmed death-ligand 1; SqCC, squamous cell carcinoma; TILes, tumor infiltrating lymphocyte enrichment score.

## Discussion

In this study, we developed a radiomic model that predicts TIL enrichment of corresponding tumor tissue using two radiomic features: GLV and LALGLE. The predicted TILes by CT radiomics significantly correlated with ICI outcome in the validation cohort independent of PD-L1 status. In addition, high TILes and high PD-L1 patients showed the most superior survival outcome to ICI compared with other groups.

Previous studies have demonstrated the possibility of a radiomics approach in the development of a biomarker for the favorable outcome of ICI (11–13). We developed a more precise radiomics model that focuses specifically on the spatial information within NSCLC and predicts TIL enrichment of corresponding tumor tissue, a potential biomarker for ICI treatment response, in collaboration with AI-powered spatial analysis of TIL by Lunit SCOPE IO (14). This approach enables us to investigate how and where exactly the TIL enrichment is visualized on the CT images and what pathophysiologic mechanism would be potentially associated, which was not discussed intensively in the previous studies.

Both GLV and LALGLE are categorized as GLSZM features, which are second-order statistical texture features. These are computed from the size-zone matrix by measuring the size of neighboring voxels with the same signal intensity (19). By grouping the adjacent same signal intensity voxels in two- or three-dimensions, a more homogeneous texture results in a wider and flatter matrix (20). Therefore, GLSZM features intensify the difference among the group of neighboring voxels with different signal intensity and have high dimensional information (21). These characteristics give GLSZM the strength to reflect intralesional texture heterogeneity which might result from the mixture of both TIL and most tumor cells.

The intralesional texture heterogeneity might have come from the several characteristics that cancer acquires during tumorigenesis, the so-called hallmarks of cancer, such as avoiding immune destruction and inducing angiogenesis (22). Such changes in cancer cells, including immunoediting to escape immunosurveillance, promotes recruitment and infiltration of various lymphocytes, resulting in complex iTME (23, 24). Hypoxia, in consequence of tumor outgrowth and insufficient vascularization, also induces regulatory T cell recruitment to promote angiogenesis and inhibit cytotoxic T cell activity (25, 26). During these processes, the tumor and its TME become more complex and each of the processes can make specific intralesional texture heterogeneity.

GLV represents the variance in gray level intensities based on GLSZM and reflects intralesional texture heterogeneity. Thus, it is reasonable that higher GLV can reflect higher TILes. This result is consistent with previous studies. Gao et al. (27) and Jeon et al. (28) reported radiomics models using GLV with a positive coefficient for predicting tumor-infiltrating regulatory T cells and cytotoxic T cells, respectively, in gastrointestinal tumors.

LALGLE and large area high gray level emphasis (LAHGLE) based on GLSZM describe the preponderance of large areas with low-density and high-density pixels in the tumor, respectively. During tumor progression, not only the necrotic low-density area but also the cellular high-density area will grow at the same time, then the value of both LALGLE and LAHGLE should be increased simultaneously. Similar results were reported by Barabino et al. (29), the tumor enlarges and the values of LAHGLE and LALGLE increase in the progressive disease of NSCLC. However, our result showed that only LALGLE was included in the model while LAHGLE was not. Considering the result, a tumor that was associated with poor TIL enrichment would have a large necrotic area compared to the size of the total tumor. It is reasonable that the proposed tumor could have a small cellular area, which is composed of both tumor and TILs.

Notably, the model used to predict TILes did not have parameters representing size, which is currently the single parameter for physicians to determine the response of tumors (30). However, responding tumors often show no change or even increase in size, so-called pseudo-progression, partly due to the enrichment of TILs after ICI administration (30, 31). Such phenomenon makes it difficult for the physician to make the best decision for the patient, but repeated biopsy is not performed routinely. Therefore, additional information using radiomic features that are associated with TILs but not the size would be helpful. Here, we have demonstrated the radiomic features associated with TILes on the pretreatment images of a single time point, it would be valuable to evaluate the temporal heterogeneity of the predicted TILes throughout the course of ICI treatment in a further study.

There are several limitations in this study. First, this is a retrospective study trained with a limited number of patients and images. This is partly because of the strict inclusion criteria that required the date of the specimen and image acquisition to be within 1 month to ensure that the iTME status of CT images and H&E slides match. The number of patients in the validation cohort was also limited which resulted in a limited significance in the subgroup analyses. However, since significant correlations with clinical findings were demonstrated, this study still showed the possibility that some of the radiomic features represent the pathophysiologic process of iTME. Second, the training and the validation were performed only in primary lung cancer, not in metastatic tumors. We restricted the tissue to the primary lesions because the surrounding attenuations at the metastasis of other organs in the images would affect some of the radiomic features (32). Further studies on the validity of our predicted TILes in the other organs are needed, especially to evaluate spatial heterogeneity. Third, the model predicted TIL with only two radiomic features which could have limited the performance for prediction of exact TILes values and made dependent on the values of two radiomic features. However, the LASSO prediction works well for any degree of correlation (33), and indeed the prediction model with 2 features was enough to predict the association of TILes with clinical outcomes of patients who received immune checkpoint inhibitors in the validation cohort, suggesting that radiomic model reflect TILs in a general way that can be applied to the other clinical cohorts. Even though, our model should be interpreted as a potential emerging biomarker that reflect part of the complex iTME that requires further validation. The further validation would include the validation on the association of the radiomic model with tissue samples and tissue biomarkers, especially in the advanced cancer patients.

In conclusion, we found the radiomic features that predict TILes were significantly associated with the outcomes of ICI. Further study is warranted to develop a model based on the radiomic feature model in this study and apply it to an exploration of the temporal and spatial heterogeneity of the tumors in clinical practices.

## Data availability statement

The raw data supporting the conclusions of this article will be made available by the authors, without undue reservation.

## Ethics statement

The studies involving human participants were reviewed and approved by the institutional review board (IRB) of Samsung Medical Center (IRB number: 2021-04-196). The ethics committee waived the requirement of written informed consent for participation.

## Author contributions

C-YO. and HYL designed the study. CP, DYJ, C-YO, and HYL had directly accessed and verified the underlying data. CP, DYJ, YC, YJO, JK, C-YO, and HYL analyzed and interpreted the data. CP, DYJ, C-YO, and HYL developed the model. DYJ, YC, YJO, and JK performed the CT image and radiomic feature extractions. CP, JR, KP, S-HL, and C-YO performed the TIL enrichment assessments. CP, DYJ, YC, S-HL, C-YO, and HYL collected and analyzed the clinical data. CP, DYJ, C-YO and HYL mainly wrote the manuscript, and all authors edited the manuscript.
