# Supplementary material for: Tumor-infiltrating lymphocyte enrichment predicted by CT radiomics analysis is associated with clinical outcomes of non-small cell lung cancer patients receiving immune checkpoint inhibitors
Source: Front Immunol. 2023 Jan 5;13:1038089. doi: 10.3389/fimmu.2022.1038089 (PMC9844154; doi:10.3389/fimmu.2022.1038089)
Supplement: Supplementary file 1 [file Presentation_1.pdf]

SUPPLEMENTARY MATERIALS

Supplementary Methods

Supplementary Methods 1. Development of Lunit SCOPE IO, artificial intelligence (AI)-powered TIL analyzer

The current version of Lunit SCOPE IO used for this study contains updated versions of the cell detection AI model and tissue segmentation AI model, compared to the versions described in our previous study[1]. This section describes the differences in relation to the previous models.

Cell Detection Model

The cell detection model detects the location of lymphocytes and tumor cells. This model is based on a convolutional neural network (CNN). In the previous model, a Faster R-CNN [2] architecture was used. In contrast, in the current version, a DeepLabV3+ [3] architecture with a Resnet-34 [4] backbone as a feature extractor was used. With this model, we posed the detection as a dense pixel prediction problem. To train the network, a circle of radius 0.95 μm centered in the point annotation of each cell is drawn, with the class value associated with the cell at that location (lymphocyte or tumor cell). The training patches cover an area of $1.5*10^5$ μm^2 (linearly resized to an image of 2048 × 2048 pixels). In each training step, a random portion of the images sized 1024 × 1024 pixels is cropped; this image is passed through on-the-fly data augmentation. The model outputs probability maps of 256 × 256 pixels, which are linearly interpolated to match the original input dimensions (1024 × 1024), therefore having a 1-to-1 pixel correspondence with the pixel annotations. Since the model predicts the likelihood of the cells being at each pixel, a post-processing stage is required to extract the locations of cells. In this stage, the likelihood maps are Gaussian filtered (σ=3), followed by local maxima detection with a radius of 0.57 μm. The model was optimized using Adam optimizer [5] with a learning rate of 0.002, decayed by a multiplicative factor of 0.2 when the validation loss does not decrease for a period of 5 epochs, and mini-batches of 24 samples.
The performance of this model in detecting lymphocytes and tumor cells in terms of F1-score is 0.68 and 0.74, respectively.

Tissue Segmentation Model

The AI model is similar to the previous version, except the backbone for feature extraction is ResNet-34[4]. Similarly, it determines if a pixel belongs to a cancer area, cancer stroma, or background regions. The model is trained with patches that cover an area of $6.1*10^5$ μm^2 and are linearly resized to images of 1024 × 1024 pixels. The model outputs probability maps of 256 × 256 pixels, which are linearly interpolated to match the original input dimensions (1024 × 1024), therefore having a 1-to-1 pixel correspondence with the pixel

annotations. In this version, there is no balanced sampling between the patches of different types of organs. The segmentation model was optimized using Adam optimizer [5] with a learning rate of 0.0001, decayed by a multiplicative factor of 0.2 when the validation loss does not decrease for a period of 6 epochs, and mini-batches of 32 samples.

The performance of this model segmenting Cancer Area and Cancer Stroma in terms of the Intersection-over-Union metric is 0.82 and 0.67, respectively.

Datasets for developing the AI models

The whole-slide images (WSI) that compose the data used for developing the previously described AI models include the following cancer primary origins: adc_nos, biliary tract, breast, colorectum, esophagus, head & neck, kidney, liver, lung, melanoma, ovary, pan-urinary, pancreas, prostate, sqcc_nos, stomach, uterine cervix, uterine endometrium, and others of unknown origin.

The *Cell Detection Model* was developed with patches extracted from 3,333 WSIs (N=2,485 for training and N=849 for validation). From these WSIs, 5,698 and 1,925 patches of area $1.5*10^5$ μm^2 per patch were extracted for training and validation, respectively. The total number of annotated cells can be found in Table 1.

Table 1 - Number of annotated cells in the training and validation sets.

	Lymphocyte	Tumor cell
Training	465,778	1,644,697
Validation	150,041	567,781

The *Tissue Segmentation Model* was developed with patches extracted from 14,807 WSIs (N=13,958 for training and N=849 for validation). From these WSIs, 55,325 and 13,958 patches of area $6.1*10^5$ μm^2 per patch were extracted for training and validation, respectively. The total area of annotated tissues can be found in Table 2.

Table 2 - Area of annotated tissues in the training and validation set.

	Area (mm^2)		
	Cancer Area	Cancer Stroma	Background
Training	7,861	5,693	18,505
Validation	600	421	983

Supplementary Methods 2. Computed tomography (CT) acquisition and 3D segmentation

All CT studies of the training cohort and the validation cohort were performed using the following parameters: the tube peak potential energy ranged from 120 kVp, the tube current ranged from 170 to 200 mA, and the scanning matrix was 512 × 512 pixels. The image data were reconstructed using standard algorithms and reformatted with a section thickness of 2.5 mm for transverse images. All CT studies were obtained at the portal venous phase (35 to 40 seconds after injection of contrast medium) using 16 or 64 multi-channel CT.

Target lesions were segmented by drawing a region of interest (ROI) with a semiautomatic approach using the commercial software AVIEW COPD (version 1.1.38.6, Coreline soft., Seoul, South Korea). The boundary of the lesion was manually modified to avoid adjacent air, fat, blood vessels, and surrounding organs. All 3D segmentations were completed by one technician and one radiologist (Y.J.O. with 5 years of experience for 3D segmentation and D.Y.J. with 6 years of experience for chest CT interpretation) and reviewed by one radiologist (H.Y.L. with 17 years of experience for chest CT interpretation). The reproducibility of radiomic characteristics was quite good. The intraclass correlation coefficient (ICC) values ranged from 0.607 to 1.000.[6]

References

1 Park S, Ock C-Y, Kim H, et al. Artificial Intelligence–Powered Spatial Analysis of Tumor-Infiltrating Lymphocytes as Complementary Biomarker for Immune Checkpoint Inhibition in Non–Small-Cell Lung Cancer. *Journal of Clinical Oncology* 2022**:** JCO-21.

2 Ren S, He K, Girshick R, Sun J. Faster r-cnn: Towards real-time object detection with region proposal networks. *Advances in neural information processing systems* 2015; **28**.

3 Chen L-C, Zhu Y, Papandreou G, Schroff F, Adam H. Encoder-decoder with atrous separable convolution for semantic image segmentation**:** 801-18.

4 He K, Zhang X, Ren S, Sun J. Deep Residual Learning for Image Recognition. *2016 IEEE Conference on Computer Vision and Pattern Recognition (CVPR)* 2016**:** 770-78.

5 Kingma DP, Ba J. Adam: A method for stochastic optimization. *Proceedings of the 3rd International Conference on Learning Representations (ICLR 2015)* 2015.

6 Lee G, Park H, Lee HY, et al. Tumor Margin Contains Prognostic Information: Radiomic Margin Characteristics Analysis in Lung Adenocarcinoma Patients. *Cancers* 2021; **13:** 1676.

Supplementary Figures

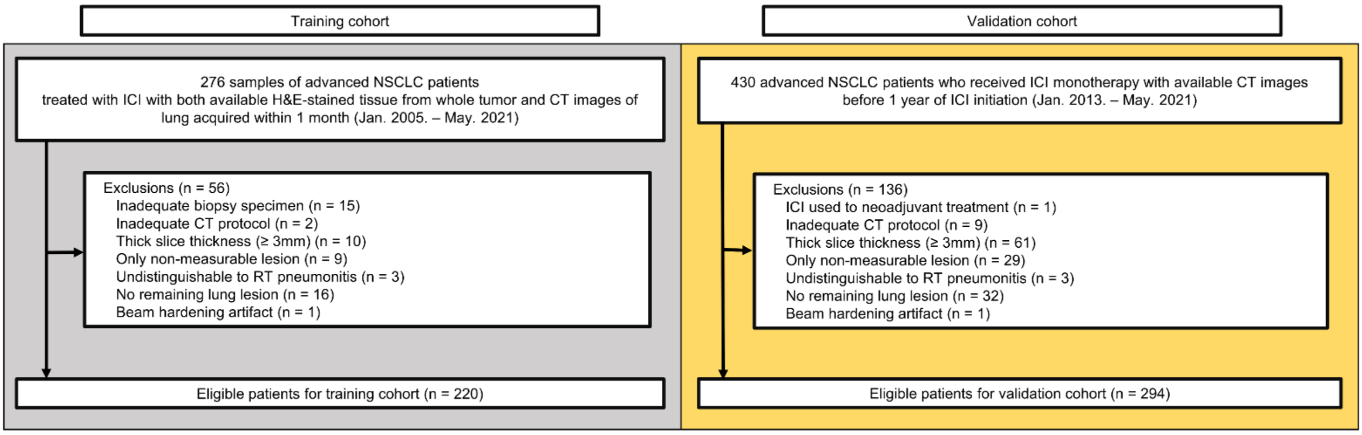

Supplementary Figure 1. Flow chart of the samples included and excluded in the study

Supplementary Figure 2

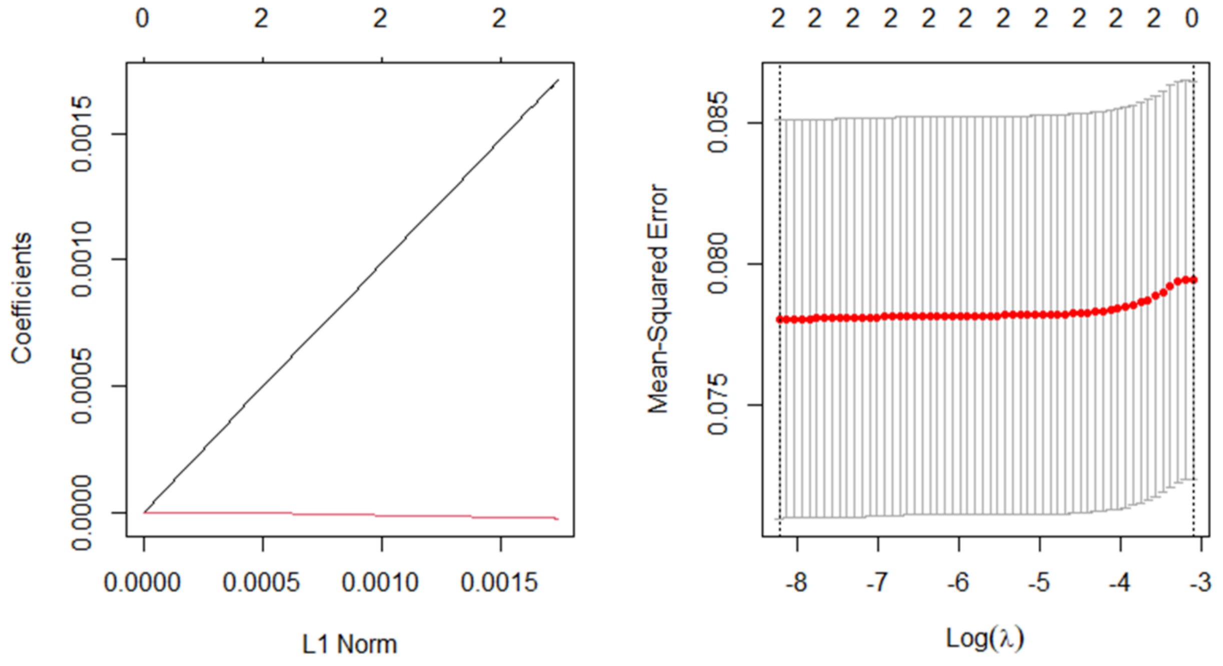

Supplementary Figure 2. The visualization of coefficients of LASSO modeling predicting TILes with gray level variance and large area low gray level emphasis

The left plot shows the path of the coefficients against the L1 norm as λ varies in the LASSO regression model. The right plot shows the cross-validation curve along the λ sequence.

Supplementary Figure 3

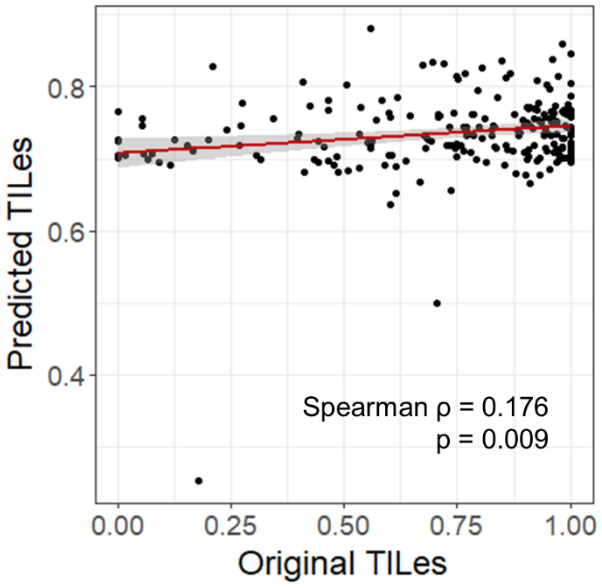

Supplementary Figure 3. Correlation of the original and predicted tumor infiltrating lymphocyte enrichment score (TILes) in the training cohort

Supplementary Figure 4

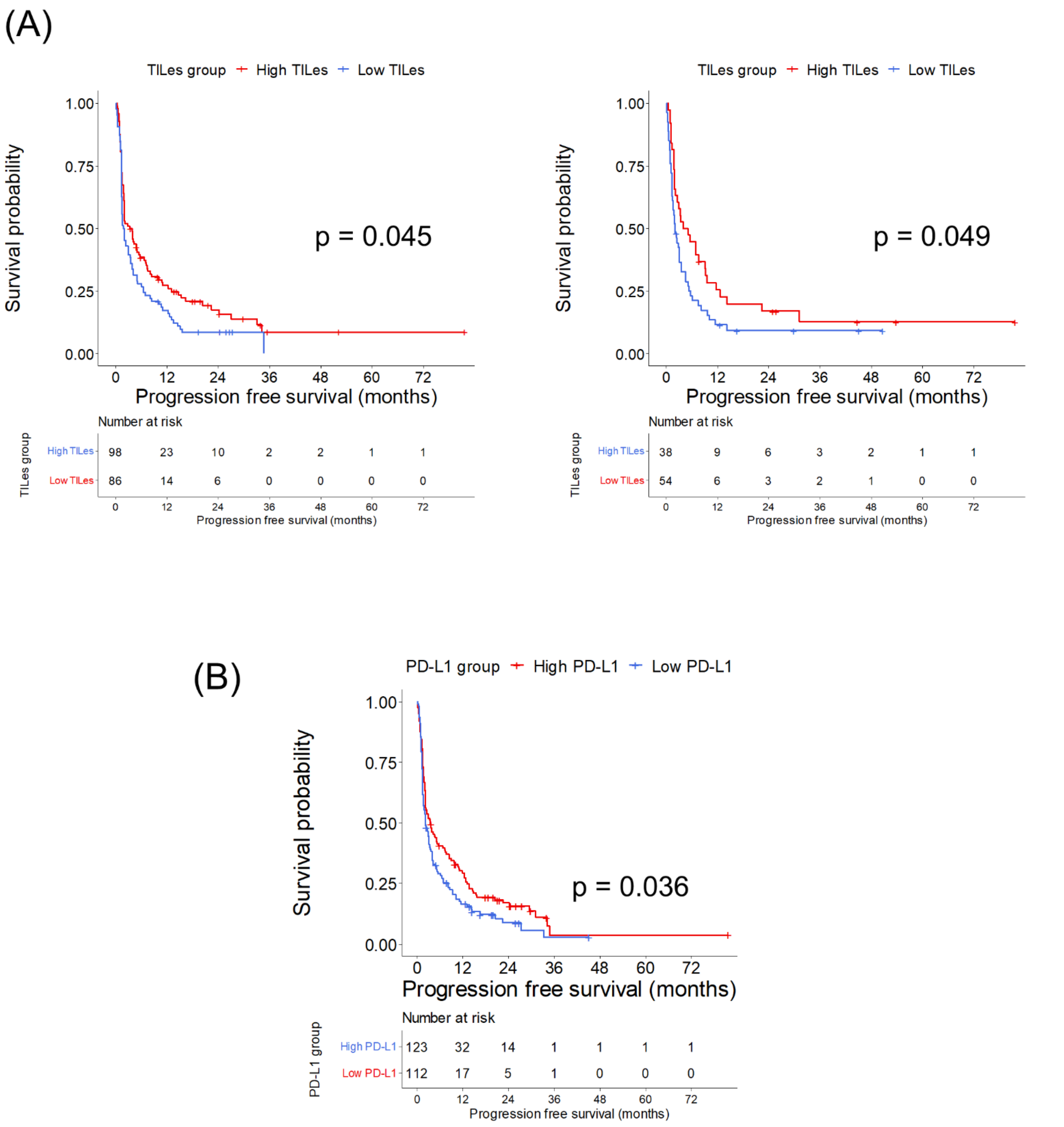

Supplementary Figure 4. Subgroup analysis of progression free survival (PFS)

(A) Kaplan-Meier curves showing PFS according to tumor infiltrating lymphocyte enrichment score (TILes) status. The left plot shows patients with adenocarcinoma and the right plot shows patients with squamous cell carcinoma. (B) Kaplan-Meier curves showing PFS according to tissue PD-L1 status. The red line represents high PD-L1, and the blue line represents the low PD-L1 group. The censored data are marked with vertical lines. The numbers at risk are provided below.
